# Association of androgen receptor expression with glucose metabolic features in triple-negative breast cancer

**DOI:** 10.1371/journal.pone.0275279

**Published:** 2022-09-30

**Authors:** Reeree Lee, Han-Byoel Lee, Jin Chul Paeng, Hongyoon Choi, Wonseok Whi, Wonshik Han, Ju Won Seok, Keon Wook Kang, Gi Jeong Cheon

**Affiliations:** 1 Department of Nuclear Medicine, Chung-Ang University Hospital, Chung-Ang University College of Medicine, Seoul, Republic of Korea; 2 Department of Nuclear Medicine, Seoul National University College of Medicine, Seoul, Republic of Korea; 3 Department of Surgery, Seoul National University College of Medicine, Seoul, Republic of Korea; 4 Biomedical Research Institute, Seoul National University Hospital, Seoul, Republic of Korea; 5 Cancer Research Institute, Seoul National University, Seoul, Republic of Korea; IRCCS Ospedale Policlinico San Martino, Genova, Italy, ITALY

## Abstract

**Background:**

Androgen receptor (AR) is a potential therapeutic target in triple-negative breast cancer (TNBC). We aimed to elucidate the association of AR expression with glucose metabolic features in TNBC.

**Methods:**

Two independent datasets were analyzed: FDG PET data of our institution and a public dataset of GSE135565. In PET analysis, patients with TNBC who underwent pretreatment PET between Jan 2013 and Dec 2017 were retrospectively enrolled. Clinicopathologic features and maximum standardized uptake value (SUV_max_) of tumors were compared with AR expression. In GSE135565 dataset, glycolysis score was calculated by the pattern of glycolysis-related genes, and of which association with SUV_max_ and AR gene expression were analyzed.

**Results:**

A total of 608 female patients were included in the PET data of our institution. SUV_max_ was lower in AR-positive tumors (*P* < 0.001) and correlated with lower AR expression (*rho* = –0.26, *P* < 0.001). In multivariate analysis, AR was a deterministic factor for low SUV_max_ (*P* = 0.012), along with other key clinicopathologic features. In the GSE135565 dataset, AR expression also exhibited a negative correlation with SUV_max_ (*r* = –0.34, *P* = 0.001) and the glycolysis score (*r* = –0.27, *P* = 0.013).

**Conclusions:**

Low glucose metabolism is a signature of AR expression in TNBC. It is suggested that evaluation of AR expression status needs to be considered in clinical practice particularly in TNBC with low glucose metabolism.

## Introduction

Triple-negative breast cancer (TNBC) is defined as breast cancer without detectable estrogen receptor (ER), progesterone receptor (PR), and human epidermal growth factor receptor 2 (HER2)/neu gene overexpression [1]. Although TNBC comprises different disease entities, it is associated with poor outcomes because of aggressive features of cancer cells and absence of effective targeted therapy [2–4]. Androgen receptor (AR) is deemed to be associated with tumorigenesis in the breast. However, AR-mediated androgenic stimulation has diverse effects in growth of breast cancer cells [5] and the role of AR in breast cancer progression is unclear. Novel treatment targets have been investigated in TNBC, and AR was recently suggested as a potential therapeutic target and a marker for subgrouping of the disease. Recent studies demonstrated that AR-negative TNBC is related to poor disease-free survival and overall survival [6–9] which demonstrates that AR can be a prognostic marker in TNBC. Additionally, AR can be a target of specific treatment and there are several ongoing clinical trials of anti-androgenic agents for breast cancers expressing [10–12].

Glucose metabolism of cancer can be easily evaluated by ^18^F-fluorodeoxyglucose (FDG) positron emission tomography (PET)/computed tomography (CT) in clinical practice. Glucose metabolism is one of the key characteristics and a significant prognostic marker in many cancers. Thus, the association of glucose metabolism with a specific gene expression or mutation has been investigated regarding crucial genes, such as EGFR or ALK [12, 13]. Although little is known about the exact mechanism, AR is presumed to be related to glucose metabolism in cancer cells, as well as normal tissues [14, 15]. In case of prostate cancer, most cancer cells show enhanced AR expression [16], and AR-driven gene expression enhances β-oxidation of fatty acid (FA) to supply energy source and ATP, and finally, reduces glucose consumption of tumor cells [17, 18]. In clinical setting, the association between AR expression and glucose metabolism in TNBC has not been investigated. Because luminal AR (LAR) subtype of TNBC was reported to show increased lipid metabolism such as FA oxidation [6], we hypothesized that AR expression would affect glucose metabolism in TNBC.

The present study aims to evaluate the association of AR with glucose metabolic features in TNBC by analyzing image phenotypes from patient data of our institution and gene expressions in a public dataset.

## Materials and methods

### Patients for FDG PET

**Fig 1:**
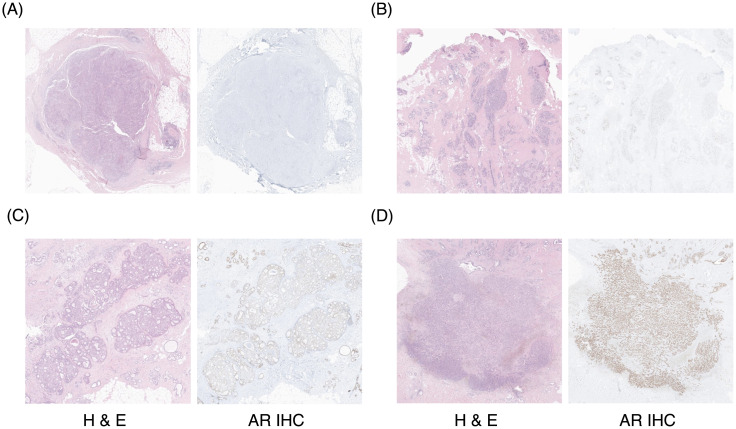
Representative immunohistochemistry images of AR expression. (A) AR expression < 1%, (B) 3%, (C) 65%, (D) 90%.

Clinicopathologic data for patients who received surgery for TNBC between January 2013 and December 2017 were retrospectively retrieved from the patient archive of our institution. The inclusion criteria were; baseline FDG PET/CT before surgical resection and any neoadjuvant treatment, and available clinicopathologic data including TNBC and AR status of surgical specimens. Evaluation of ER, PR, HER2, and AR expression for pathologic specimens were routinely performed. Cutoff values for estrogen receptor and progesterone receptor positivity were determined as ≥ 1% on immunohistochemistry, according to the current guidelines [19]. HER2 status was evaluated according to the ASCO/CAP guideline [20], using 4B5 Ventana assay and PathVysion HER-2 DNA probe Kit (Vysis). Immunohistochemistry for AR was carried out from each paraffin block, applying the specific antibody (clone SP107, Rabbit Monoclonal, Roche Diagnostics; ready to use) on automated system (Ventana Benchmark Ultra), according to the manufacturer’s instructions. Representative immunohistochemistry images of various AR expressions are shown in Fig 1. The cutoff value for AR positivity was determined as ≥ 1%. Information on clinicopathologic features such as age, tumor size, lymph node status, pathologic stage, and histologic grade was obtained from medical records review.

The protocol of this retrospective study was approved and informed consent from each patient was waived by the Institutional Review Board of Seoul National University Hospital (IRB No.: 1905-136-1035). All methods were performed in accordance with the relevant guidelines and regulations.

### PET image acquisition and analysis

All patients fasted for at least 6 hours, and blood glucose levels were confirmed to be < 140 mg/dL. 5.18 MBq/kg (0.14 mCi/kg) of ^18^F-FDG was intravenously injected, and PET/CT was performed 60 minutes after injection using dedicated PET/CT scanners (Biograph mCT40 or mCT64, Siemens Healthcare). A low-dose CT scan (120 kVp, 50 mAs) was performed first for attenuation correction and anatomical localization, and PET images were obtained from the skull base to the proximal thigh for 1 minute per bed position (6–7 bed positions for a patient). PET images were reconstructed by an iterative algorithm (ordered subset expectation maximization, iteration 2, subset 21). Images were reviewed by two specialists (J.C.P. with 20-year experience and R.L. with 5-year experience), who were unaware of patient and clinical information. On PET/CT fusion images, a volume of interest was drawn carefully to encircle the primary breast tumor and SUV_max_ was measured using an analysis software package (Syngo.via, Siemens Healthcare) as the index for glucose metabolism.

### GEO GSE135565 gene expression dataset analysis

The correlations between SUV, gene expressions for glucose metabolism and AR were additionally tested using a public dataset, the Gene Expression Omnibus (GEO) Series, GSE135565 (https://www.ncbi.nlm.nih.gov/geo/download/?acc=GSE135565). This dataset includes clinical, microarray, and SUV_max_ data of 84 TNBC patients [21]. Glycolysis signatures were analyzed by using the single sample gene set enrichment analysis (ssGSEA) [22, 23] and metabolic pathway gene information obtained from the Reactome database [24]. ssGSEA for glycolysis score was performed by the Gene Set Variation Analysis (GSVA) package of R/Bioconductor [25]. The glycolysis score (enrichment score) was calculated from the REACTOME_GLYCOLYSIS [26], in which the glycolysis score indicates how close gene expression pattern of a sample is, to expect expression pattern of the glycolysis-related gene set (S1 Table). The enrichment score of FA β-oxidation was calculated from the REACTOME_MITOCHONDRIAL_FATTY_ACID_BETA_OXIDATION [27].

### Statistical analysis

Values were expressed as mean ± standard deviation (SD) for parametric test or median [interquartile range] for non-parametric test. Chi-square test was used for comparison of AR positivity according to the clinical characteristics. Mann-Whitney U test were used for comparison of SUV_max_ according to the various clinicopathological features. Tumors were classified into high and low SUV_max_ groups using the median SUV_max_ as a cutoff value, and univariate and multivariate logistic regression analyses were performed to select significant clinicopathologic factors for determining tumors of high SUV_max_. Pearson’s correlation test was performed to evaluate correlations among AR, SUV_max_, and glycolysis-associated genes expressions. SUV_max_ and AR expression of our institution dataset were analyzed using Spearman correlation test, because of non-normality. Data were analyzed using the R program (ver. 3.4.5). *P*-values less than 0.05 were deemed to be statistically significant.

## Results

### Patients for FDG PET analysis

A total of 608 female patients (age 54.2 ± 11.7 y, range 26–93 y) were included in the analysis. Most of the cases were invasive ductal carcinoma (534 patients, 87.8%), and 473 cases (77.8%) were histological grade III. Lymph node metastasis was present in 314 patients (51.6%). Tumor size was larger than 2.0 cm in 402 patients (66.1%). Patient characteristics are summarized in Table 1. Among 608 cases, 216 (35.5%) were AR-positive, and the other 392 (64.5%) were AR-negative. The rate of AR positivity was higher in old age (> 50 years, *P* = 0.001) and low histologic grade (grade I/II, *P* < 0.001) (Table 2). There was no significant difference in AR positivity according to tumor size, lymph node metastasis, and overall cancer stage.

**Table 1:** Patient characteristics.

Characteristics	N (%)
Age	
≤ 50	238 (39.1%)
> 50	370 (60.9%)
Tumor stage	
I	152 (25.0%)
II	274 (45.1%)
III	163 (26.8%)
IV	19 (3.1%)
T-stage	
T1	194 (31.9%)
T2	334 (54.9%)
T3	40 (6.6%)
T4	40 (6.6%)
N-stage	
N0	294 (48.4%)
N1	154 (25.3%)
N2	101 (16.6%)
N3	59 (9.7%)
Pathologic subtypes	
Ductal	534 (87.8%)
Lobular	5 (0.8%)
Metaplastic	44 (7.2%)
Others	25 (4.2%)
Histologic grade	
I	4 (0.7%)
II	127 (21.0%)
III	473 (77.8%)

**Table 2:** Androgen receptor expression according to clinicopathologic characteristics.

Characteristic	AR positivity, N (%)	P
Overall	216/608 (35.5%)	
Age		0.001
≤ 50	66/238 (27.7%)	
> 50	150/370 (40.5%)	
Tumor size		0.064
≤ 2 cm	84/206 (40.8%)	
> 2 cm	132/402 (32.8%)	
Lymph node metastasis		0.304
Negative	111/294 (37.8%)	
Positive	105/314 (33.4%)	
Stage		1.000
I/II	151/426 (35.4%)	
III/IV	65/182 (35.7%)	
Histologic grade		< 0.001
I/II	72/131 (55.0%)	
III	142/473 (30.0%)	

AR: androgen receptor

### Association of AR expression with FDG PET findings

**Fig 2:**
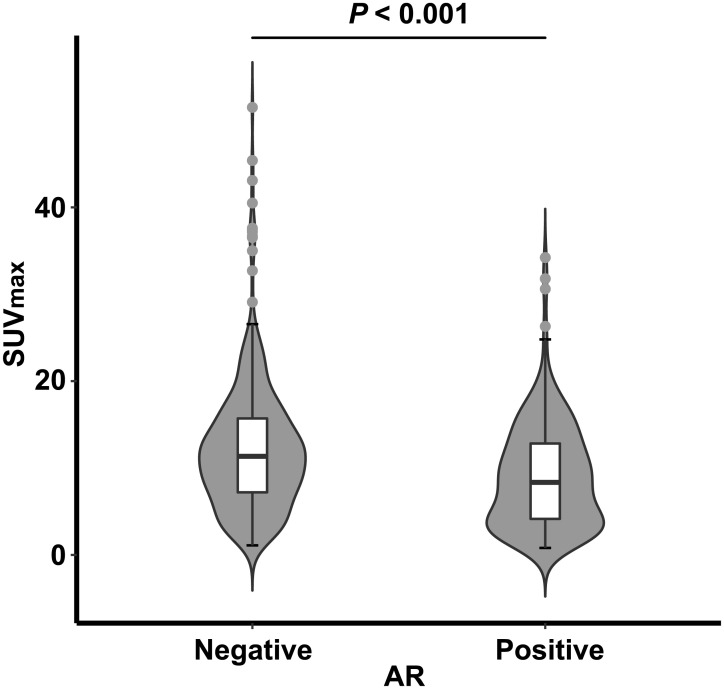
Difference of SUV_max_ according to AR status in FDG PET analysis. SUV_max_ is higher in AR-negative than in AR-positive TNBC. Violin plots indicate the distribution of SUV_max_ according to the AR positivity. Box plot inside the violin plot denotes median value as a central line within a box, 25 percentile and 75 percentile value as an outline of a box and minimum and maximal number are marked as error bar with outliers marked separately. AR, androgen receptor expression on immunohistochemistry.

Almost all breast cancer lesions showed high glucose metabolism and average SUV_max_ was 11.0 ± 7.1 (range 0.8–51.5). SUV_max_ was significantly higher in tumors of large size, positive lymph node metastasis, high histologic grade, and high Ki-67 index (*P* < 0.001 for all in Mann-Whitney U test, Table 3). SUV_max_ was also significantly higher in AR-negative tumors than in AR-positive tumors (*P* < 0.001, Fig 2 and Table 3). There was a weak but significant negative correlation between SUV_max_ and the degree of AR positivity (*rho* = –0.26, *P* < 0.001 in Spearman test). SUV_max_ was significantly different between AR-positive and AR-negative tumors, regardless of cutoff values for AR positivity (S2 Table). In a multivariate analysis to select independent factors for determining tumors of low SUV_max_ (cutoff 10.3, median value of SUV_max_), AR expression was selected as a significant factor for determining SUV_max_ (*P* = 0.012), along with tumor size (*P* < 0.001), presence of lymph node metastasis (*P* < 0.001), and histologic grade (*P* < 0.001) (Table 4). In additional analysis using a cutoff of 10% for AR positivity, AR was still a significant determinant of SUV_max_ (S3 Table).

**Table 3:** FDG uptake according to various clinicopathologic factors.

Factors	N	Median SUV_max_ [interquartile range]	*P*
Age			0.002
≤ 50	238	11.0 [7.2–16.1]	
> 50	370	9.7 [5.2–13.8]	
Tumor size (cm)			< 0.001
≤ 2.0	206	5.8 [3.3–10.0]	
> 2.0	402	12.3 [8.5–16.1]	
Lymph node metastasis			< 0.001
Positive	314	12.0 [8.3–16.1]	
Negative	294	8.2 [4.0–12.4]	
Stage			< 0.001
I/II	426	8.9 [4.8–13.4]	
III/IV	182	12.8 [9.3–17.0]	
Histologic grade			< 0.001
I/II	131	6.4 [3.3–11.5]	
III	473	11.1 [7.2–15.7]	
Ki-67			< 0.001
High	264	11.4 [7.3–16.1]	
Low	333	9.4 [4.7–13.5]	
AR expression			< 0.001
Positive	216	8.4 [4.1–12.9]	
Negative	392	11.4 [7.2–15.7]	

AR, androgen receptor; SUV_max_, maximum standardized uptake value

**Table 4:** Univariate and multivariate analyses for determining SUV_max_.

Variables	Univariate analysis		Multivariate analysis (backward deletion)	
	Odds ratio (95% CI)	*P*	Odds ratio (95% CI)	*P*
Age > 50 y vs. ≤ 50 y)	0.67 (0.49–0.94)	**0.0184**	eliminated	NA
Tumor size (> 2 cm vs. ≤ 2 cm)	5.27 (3.62–7.68)	**< 0.001**	4.0 (2.65–6.03)	**< 0.001**
Lymph node metastasis (positive vs. negative)	2.94 (2.11–4.09)	**< 0.001**	2.07 (1.42–3.02)	**< 0.001**
Histologic grade (III vs. I/II)	2.62 (1.74–3.95)	**< 0.001**	2.38 (1.50–3.80)	**< 0.001**
Ki-67 (> 15 vs. ≤ 15)	1.72 (1.24–2.38)	**0.001**	1.36 (0.93–1.97)	0.112
AR expression (≥ 1% vs. < 1%)	0.49 (0.35–0.68)	**< 0.001**	0.61 (0.41–0.90)	**0.012**

CI, confidence interval; AR, androgen receptor; SUV_max_, maximum standardized uptake value; Bold p value: statistically significant (p < 0.05) on logistic regression analyses

### Association between expressions of AR, glycolysis, and FA metabolism-related genes

**Fig 3:**
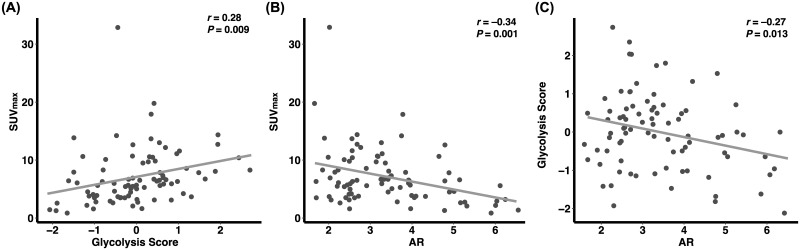
Correlations among AR, SUV_max_, and glycolysis-associated genes expressions analyzed using GSE135565 database. SUV_max_ was well correlated with glycolysis score (A). A negative correlation between AR expression and SUV_max_ was observed (B). The AR expression is inversely correlated with glycolysis score (C). AR, expression level of androgen receptor gene; Glycolysis score indicates how close gene expression pattern of a sample is, to expected expression pattern of the glycolysis-related gene set.

**Fig 4:**
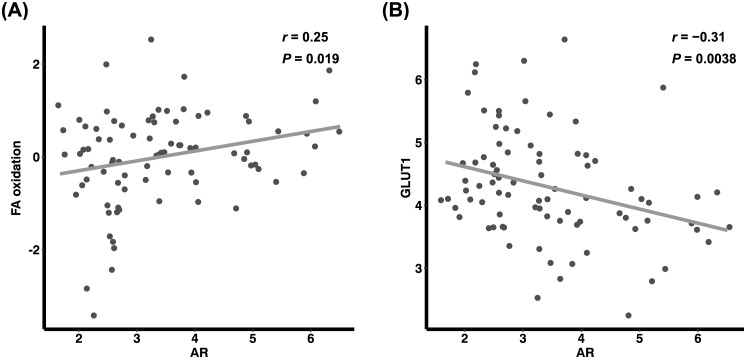
Correlations of AR with expression of FA β-oxidation-associated genes and GLUT1. AR expression show positive correlation with FA oxidation (A) and negative correlation with GLUT1 expression (B). AR, expression level of androgen receptor gene; FA, fatty acid; FA oxidation, gene set enrichment score of FA β-oxidation-associated genes; GLUT1, expression level of GLUT1 (SLC2A1) gene.

In the analysis of the GSE135565 dataset, glycolysis score exhibited a significant correlation with SUV_max_ of tumors (*r* = 0.28, *P* = 0.009, Fig 3A). In accordance with the results from our patient data analysis, there was a significant negative correlation between SUV_max_ and AR expression (*r* = –0.34, *P* = 0.001, Fig 3B). Additionally, AR expression also exhibited a significant negative correlation with glycolysis score (*r* = –0.27, *P* = 0.013, Fig 3C). AR expression showed a significant positive correlation with enrichment score of FA β-oxidation (*r* = 0.25, *P* = 0.019, Fig 4A), and a negative correlation with GLUT1 (SLC2A1) expression (*r* = –0.31, *P* = 0.004, Fig 4B).

## Discussion

In this study, it was demonstrated that AR expression is consistently related to low glucose metabolic features. In our patient data, SUV_max_ was significantly higher in AR-negative tumors than in AR-positive tumors. Additionally, in both datasets, negative correlations were observed between SUV_max_ and AR expression. In GSE135565 dataset, AR expression also exhibited a negative correlation with the glycolysis score, which is determined by the expression pattern of glycolysis-related genes.

TNBC exhibit aggressive features and poor outcomes [28]. The poor outcome is caused by innate biological features of TNBC, as well as lack of response to common targeted therapies, including anti-hormonal and anti-HER2 agents. FDG PET is a well-known imaging biomarker for glucose metabolic activity, which is related to aggressive features of cancers and poor prognosis. In most cancers, high glucose metabolic activity and high SUV_max_ on FDG PET is a significant marker for poor prognosis. Breast cancers with high glucose metabolism also have poor prognosis, and TNBC tends to exhibit higher SUV_max_ than other types of breast cancer [29].

AR is a member of the steroid hormone receptor family, and there are emerging pieces of evidence on the associations between androgen effect and tumorigenesis in breast cancer [30]. Recently, the subclassification of TNBC by AR expression has been suggested [7–9, 31–33]. In the current study, AR was positive in 35.4% of TNBC, and related to young age and low histologic grade of tumors, which is in accordance with previous studies [7, 31–33].

Currently, there is not much information on the relation between AR status and glucose metabolism. Humbert *et al*. reported that there is a trend that TNBC with positive AR exhibits lower FDG uptake than those with negative AR, in a small study, including 50 TNBC patients [34]. However, statistical significance was not reached in the study, probably due to the small case number. In our analysis of 608 patients, FDG uptake was significantly lower in AR-positive TNBC. AR was an independent factor for determining low SUV_max_ in multivariate analysis, including other key pathologic factors. The association between FDG uptake and AR status was not affected by varying cutoff value for AR positivity. The results suggest that AR negativity is independently related to aggressive biological features and poor prognosis in TNBC. GSE135565 dataset was additionally analyzed in the present study as a crosscheck for the association between AR expression and glucose metabolism at the gene level. In the analysis, there was a negative correlation between AR expression and SUV_max_, which is in agreement with the results from our patient data. Furthermore, a negative correlation was observed between AR and glycolysis score, which suggests that AR expression decreases the glucose metabolism at the gene and cellular level in TNBC. In this dataset, glycolysis score was also well correlated with the image phenotype of SUV_max_.

Many studies have reported that positive AR expression is a good prognostic marker and is related to favorable clinical outcomes in breast cancer [7–9, 32, 35]. The findings of the present study also suggest that AR expression is a good prognostic marker by presenting lower glucose metabolism, and presumably, less aggressive biological features. However, there have been some conflicting results on the prognostic effect of AR [32, 36, 37], which needs further investigations. At present, AR testing and AR-targeted therapy are not yet standard of care in TNBC patients. The recognition of low glucose metabolism in a known TNBC might allow additional immunohistochemical evaluation for AR expression and AR-targeted therapy as part of a clinical trial. Hence, another implication of our study is that AR status needs to be considered in TNBC, particularly in cases with low SUV_max_.

Specific mechanism or signaling pathway for the correlation between AR and glucose metabolism is unclear. In this study, there was a significant positive correlation between AR expression and FA β-oxidation, which is consistent with the increased lipid metabolism in LAR subtype of TNBC [38]. The shift of metabolic substrate from glycolysis to FA oxidation may be one of the causes of reduced GLUT1 expression, glucose consumption, and decrease in FDG uptake [39]. In case of prostate cancer that expresses abundant androgen receptors, it is well-known that the tumor shows low FDG avidity and its grade is not well correlated with FDG accumulation [17]. Further studies are required on the specific mechanism for the AR effect on glucose metabolism.

There are a few limitations in the present study. First, the patient image analysis was performed in a retrospectively collected cohort, and there might have been an unexpected selection bias. However, because FDG PET/CT and AR immunohistochemistry were performed without specific selection, the possible bias would have been fairly controlled. Second, the prognostic effect of AR expression and FDG uptake was not evaluated because of the insufficient follow-up time. Based on the current study, further prospective studies for the prognostic role of AR expression and FDG uptake are warranted.

## Conclusion

AR-positive TNBC tumors show low FDG uptake on PET, which is associated with low expression of glycolysis-related genes. In our multivariate analyses, AR expression was an independent determinant for low glucose metabolism. The results suggest that low glucose metabolism is a signature of AR expression in TNBC, and that AR expression status needs to be considered in clinical practice particularly in TNBC with low glucose metabolism.

## Supporting information

S1 Table Glycolysis-related genes from the REACTOME_GLYCOLYSIS. (DOCX)

S2 Table SUV_max_ according to androgen receptor status with varying cutoff values for androgen receptor positivity. AR: androgen receptor.
(DOCX)

S3 Table Univariate and multivariate analyses for determining SUV_max_ using ≥ 10% for AR positivity. AR: androgen receptor.
(DOCX)
