# Supplementary material for: Association of androgen receptor expression with glucose metabolic features in triple-negative breast cancer
Source: PLoS One. 2022 Sep 30;17(9):e0275279. doi: 10.1371/journal.pone.0275279 (PMC9524647; doi:10.1371/journal.pone.0275279)
Supplement: S1 Table — (DOCX) [file pone.0275279.s001.docx]

**S1 Table.** Glycolysis-related genes from the REACTOME_GLYCOLYSIS

| *AAAS* | *GCKR* | *NUP160* | *NUP160* | *PGAM1* | *PPP2R1A* |
| --- | --- | --- | --- | --- | --- |
| *ADPGK* | *GNPDA1* | *NUP188* | *NUP188* | *PGAM2* | *PPP2R1B* |
| *ALDOA* | *GNPDA2* | *NUP205* | *NUP205* | *PGK1* | *PPP2R5D* |
| *ALDOB* | *GPI* | *NUP210* | *NUP210* | *PGK2* | *PRKACA* |
| *ALDOC* | *HK1* | *NUP214* | *NUP214* | *PGM2L1* | *PRKACB* |
| *BPGM* | *HK2* | *NUP35* | *NUP35* | *PGP* | *PRKACG* |
| *ENO1* | *HK3* | *NUP37* | *NUP37* | *PKLR* | *RAE1* |
| *ENO2* | *NDC1* | *NUP42* | *NUP42* | *PKM* | *RANBP2* |
| *ENO3* | *NUP107* | *NUP43* | *NUP43* | *POM121* | *SEC13* |
| *GAPDH* | *NUP133* | *NUP50* | *NUP50* | *POM121C* | *SEH1L* |
| *GAPDHS* | *NUP153* | *NUP54* | *NUP54* | *PPP2CA* | *TPI1* |
| *GCK* | *NUP155* | *NUP58* | *NUP58* | *PPP2CB* | *TPR* |
