# Supplementary material for: Association of androgen receptor expression with glucose metabolic features in triple-negative breast cancer
Source: PLoS One. 2022 Sep 30;17(9):e0275279. doi: 10.1371/journal.pone.0275279 (PMC9524647; doi:10.1371/journal.pone.0275279)
Supplement: S2 Table — AR: androgen receptor. (DOCX) [file pone.0275279.s002.docx]

**S2 Table.** SUV_max_ according to androgen receptor status with varying cutoff values for androgen receptor positivity

| Cutoff | AR-negative | | AR-positive | | *P* |
| --- | --- | --- | --- | --- | --- |
|  | N (%) | SUV_max_ | N (%) | SUV_max_ |  |
| < 1% | 392 (64.5) | 12 [7.2 – 15.7] | 216 (35.5) | 8.4 [4.1 – 12.9] | < 0.001 |
| < 5% | 444 (73.0) | 11.3 [7.2 – 15.6] | 164 (27.0) | 7.0 [3.5 – 12.1] | < 0.001 |
| < 10% | 466 (76.6) | 11.3 [7.2 – 15.6] | 142 (23.4) | 6.5 [3.5 – 11.4] | < 0.001 |
| < 15% | 484 (79.6) | 11.1 [7.2 – 15.4] | 124 (20.4) | 6.4 [3.4 – 11.2] | < 0.001 |
| < 20% | 493 (81.1) | 11.0 [7.0 – 15.3] | 115 (18.9) | 6.4 [3.4 – 11.2] | < 0.001 |

AR: androgen receptor
