# Supplementary material for: Association of androgen receptor expression with glucose metabolic features in triple-negative breast cancer
Source: PLoS One. 2022 Sep 30;17(9):e0275279. doi: 10.1371/journal.pone.0275279 (PMC9524647; doi:10.1371/journal.pone.0275279)
Supplement: S3 Table — AR: androgen receptor. (DOCX) [file pone.0275279.s003.docx]

**S3 Table.** Univariate and multivariate analyses for determining SUV_max_ using a cutoff of 10% for AR positivity

| Variables | Univariate analysis | | Multivariate analysis  (backward deletion) | |
| --- | --- | --- | --- | --- |
|  | Odds ratio  (95% CI) | *P* | Odds ratio (95% CI) | *P* |
| Age  (> 50 y vs. ≤ 50 y) | 0.67  (0.49 – 0.94) | **0.0184** | eliminated | NA |
| Tumor size  (> 2 cm vs. ≤ 2 cm) | 5.27  (3.62 – 7.68) | **< 0.001** | 3.86  (2.56 – 5.83) | **< 0.001** |
| Lymph node metastasis  (positive vs. negative) | 2.94  (2.11 – 4.09) | **< 0.001** | 2.10  (1.44 – 3.07) | **< 0.001** |
| Histologic grade  (III vs. I/II) | 2.62  (1.74 – 3.95) | **< 0.001** | 2.30  (1.44 – 3.68) | **< 0.001** |
| Ki-67  (> 15 vs. ≤ 15) | 1.72  (1.24 – 2.38) | **0.001** | 1.35  (0.93 – 1.97) | 0.116 |
| AR expression  (≥ 10% vs. < 10%) | 0.35  (0.23 – 0.52) | **< 0.001** | 0.49  (0.31 – 0.77) | **0.002** |

CI, confidence interval; AR, androgen receptor; SUV_max_, maximum standardized uptake value; Bold p value: statistically significant (p < 0.05) on logistic regression analyses
